# Components of a Digital Storytelling Intervention for Human Papillomavirus and Cancer Prevention Among LGBTQ+ Individuals: Formative Mixed Methods Inquiry

**DOI:** 10.2196/58163

**Published:** 2025-05-30

**Authors:** Gabrielle Darville-Sanders, Dominique Munroe, Emilie Corluyan, Utibeabasi Ikoiwak, Jennifer Nguyen, Chad Mandula, Portia Thomas, Brandon Sanders

**Affiliations:** 1 Department of Public Health, Mercer University, 3001 Mercer University Drive, Atlanta, GA, 30341, United States, 1 678-547-6492; 2 College of Pharmacy, Mercer University, Atlanta, GA, United States; 3 Institute of Higher Education, University of Georgia, Athens, GA, United States; 4 School of Nursing, Yale University, West Haven, CT, United States; 5 Empowerment Resource Center, Atlanta, GA, United States

**Keywords:** human papillomavirus, vaccination, cancer prevention, digital storytelling, public health

## Abstract

**Background:**

Human papillomavirus (HPV) is one of the most prevalent sexually transmitted infections in the United States; however, vaccination uptake falls far below the goal of 80% of the population set forth by Healthy People 2030. Specifically, within the LGBTQ+ (lesbian, gay, bisexual, transgender, queer/questioning) population, HPV vaccination adherence remains a complex issue. Due to the widespread use of technology within the young adult population, digital health tools such as digital storytelling (DST) have been promoted as an effective way to increase vaccination uptake.

**Objective:**

The purpose of this study was to conduct a formative inquiry into (1) what components should be considered for inclusion in an HPV documentary tailored for sexual and gender minority populations and (2) what dissemination channels would be more effective and impact the uptake and completion of the HPV vaccine among sexual and gender minority populations. Additionally, this study aims to provide insight into perceived HPV risk and its implications on the HPV vaccine uptake within the LGBTQ+ population.

**Methods:**

A mixed methods study was conducted between January 2021 and September 2021 in Atlanta, Georgia. Intake surveys were distributed to individuals identifying as members of the LGBTQ+ community to examine demographic characteristics, barriers to vaccine adherence, and current HPV vaccination status. Perceived HPV risk was assessed using 5 statements on a 1 to 7 Likert scale. Key informant interviews were conducted via Zoom with participants who completed the intake surveys and consented to be interviewed. Transcripts were coded and analyzed using the constant comparison method for emergent themes surrounding components of effective DST campaigns.

**Results:**

Forty-seven individuals completed the intake survey and interview. A total of 13 out of 47 (27.7%) of participants indicated that they were not sure when provided with the statement “I am likely to get HPV”, whereas 12 out of 47 (29.8%) participants strongly disagreed with the statement “I am at high risk for getting HPV” and 13 out of 47 (27.7%) participants indicated that they were not sure when presented with the statement “HPV would be a serious threat to the quality of my life.” A total of 14 out of 47 (29.8%) participants responded that they were not sure to the statement “HPV would be a severe threat to my health” and 13 out of 47 (27.7%) participants strongly agreed that “HPV would be a severe threat to my sex life.” Qualitative analysis indicated a high level of stigma experienced in interactions between the LGBTQ+ population and private practitioners. Major barriers to vaccination hesitancy were concerns about age, perceived reduced risk, and lack of provider recommendation. Participant interviews revealed that “Real Outcomes,” and “Accurate Representation” were the main components that should be considered for inclusion in an HPV documentary tailored for sexual and gender minority populations.

**Conclusions:**

Creation of a DST intervention within the LGBTQ+ population should include information surrounding the real outcomes of HPV and accurate representation.

## Introduction

Vaccines have proven to be an effective primary public health measure towards preventing disease, disability, and death from infectious diseases [1-3]. Vaccination is not only vital to individual health, but also a crucial component of improving the health of communities by reducing the spread of disease. Human papillomavirus (HPV) is the most prevalent sexually transmitted infection in the United States, with a prevalence of 40% among persons aged 15‐59 years [4]. It is also responsible for a significant amount of morbidity and mortality [5,6]. Despite disease prevalence, HPV vaccination uptake has fallen short of 80% of the general population, a goal set forth by Healthy People 2030 [7].

The World Health Organization identified complacency, inconvenience, and lack of confidence as several of the various factors that can influence an individual’s intention to become vaccinated. These factors, when not addressed, result in vaccine uncertainty or delayed vaccination and the overall undermining of public health efforts due to decreased vaccine coverage and an increased risk of disease outbreaks [8]. Vaccination hesitancy is not a new problem [9-12]and has been recognized by the World Health Organization as a top 10 threat to global health [13-15]. Controversy has consistently surrounded the measles, mumps and rubella vaccine [14], diphtheria, tetanus and pertussis vaccine [14,16], swine flu vaccine [14,17], polio vaccine [14,18], influenza vaccine [19], and most recently the COVID-19 vaccine [9,13,20,21]; affecting adherence of the general population. Similarly, the HPV vaccine has also seen low levels of initial uptake and moderate levels of series completion [22,23]. In 2016, only 60% of 13‐17-year-olds completed 1 dose of the vaccine series [24], and less than 50% of teens were up to date with the recommended HPV vaccination series [4,24,25]. Furthermore, major disparities surrounding geography [26,27], economic status, educational level [28], and health care provider recommendations [29] have been found to be correlated with HPV vaccination.

Various strategies have been used to address vaccine hesitancy, such as peer mentoring [30-32] and workshops/lectures [33]. One of the more novel ideas, however, has been that of digital storytelling (DST). The participatory nature of DST has significant potential to promote participants’ psychosocial health and well-being, revealing hidden stories and initiating community dialogue about issues that are pressing and concerning to research participants [34]. Furthermore, DST as a medium can decrease the time between knowledge generation and implementation [34]. Given the high use of technology among young adults [35], digital health technologies have been touted as an effective way to encourage uptake among this group [36,37]. In DST, participants describe their personal stories to an audience that is specific to a lived experience. On the other side, the audience is influenced by the story through their own relevant feelings or responses [34]. This model has been investigated by researchers to ascertain whether or not it can be effective in regard to the distrust surrounding the COVID vaccine among Black Americans [38]. Kim et al [39] used DST as a means to promote HPV vaccination among Korean American College women and found the intervention to result in improvement in knowledge and attitude at the conclusion of the intervention. Additionally, in their study, the experimental group was twice as likely to receive the HPV vaccine as the comparison group. A similar intervention was conducted by Chen et al [40] in which they found DST to be effective in increasing Korean mothers’ intention to vaccinate their children against HPV (from 53% to 74%).

Despite the well-documented success of DST, there remain several limitations to its use. Ethical challenges can arise with the use of DST, specifically regarding the use of participant photographs and voices, which can result in issues with the maintenance of participant confidentiality [34]. There also remains a gap in the use of DST specifically in relation to the LGBTQ+ (lesbian, gay, bisexual, transgender, queer/questioning) population. There is a pattern of discrimination, stigma, and lack of awareness among health care providers, along with issues surrounding access to sex education, health care, and insurance coverage that have impeded LGBTQ+ populations from accessing health care services and thus contributed to major disparities surrounding HPV and HPV vaccination [41-47]. The purpose of this study was to conduct a formative inquiry into (1) what components should be considered for inclusion in an HPV documentary tailored for sexual and gender minority populations, and (2) what dissemination channels would be more effective and impact the uptake and completion of the HPV vaccine among sexual and gender minority populations. Thus, informing the construction of a DST intervention to increase HPV vaccine uptake within the LGBTQ+ population. Additionally, this study aims to provide insight into the influence that perception of HPV risk can have on HPV vaccine uptake.

## Methods

### Study Design

This mixed method, explanatory sequential study was conducted among individuals who identified as members of the LGBTQ+ community between January 2021 and September 2021 in Atlanta, Georgia.

### Ethical Considerations

This study was conducted according to the guidelines 2018 Federal Regulations 21 CFR 56.110(b) and 45 CFR 46.110(b) (for expedited review) and was approved under categories _6, _7 per 63 FR 60364. All procedures involving research study participants were approved by the Mercer University Institutional Review Board on April 2, 2020. Written informed consent was obtained from all participants.

On the opening page of the survey, participants were presented with information about the study’s objectives and scope, followed by an informed consent statement. Participants were free to withdraw at any point without consequence. In addition, participation was anonymous, and no personally identifiable information was collected or used. Informed consent was obtained from all participants involved in the study, in compliance with the H2004105 Mercer University Institutional Review Board protocol. Participants were compensated for their participation via a US $40 Amazon gift card for completion of both the intake survey and participant interview.

### Participants

Participants were recruited using convenience sampling to recruit participants for the study. Using statewide organizational connections, community partnerships, web-based or email advertising, and word-of-mouth recruitment, 60 participants were recruited. Eligible participants were those aged 18 years or older, currently residing in Atlanta, Georgia, capable of providing informed consent before starting the survey, and identifying as members of the LGBTQ+ community.

After enrolling in the study via SuperSaaS software (SuperSaaS B.V.), survey participants were emailed the link to the Qualtrics survey with the informed consent and intake survey to complete prior to their interview participation. Participant information was deidentified using a participant identification number assigned to all registrants via the SuperSaaS software [48]. Reminder emails were sent 1 week prior to the session, with follow-up emails sent 3 days before the interview session. Follow-up emails were sent to nonrespondents to encourage participation. If no response was received by the morning of the final day of data collection, those participants were removed from the study, resulting in a cohort of 47.

### Procedure

Demographic data were obtained from the 15 initial intake questions distributed after participants had signed up for an interview session. The intake survey was distributed via Qualtrics, took approximately 5‐10 minutes to complete, and was developed with input from a subject matter expert specializing in LGBTQ populations. Scale items were adapted from a previous HPV study assessing 3 key constructs: risk perception (3 items), self-efficacy (3 items), and behavioral intention (4 items) using a 7-point Likert scale(1=strongly disagree to 7=strongly agree) [49]. The total score was calculated from the sum of the response items, with higher scores indicating a greater perceived risk of HPV. An additional LGBTQ-related demographic question was added, but was not revalidated or pretested.

Due to special precautions associated with the COVID-19 pandemic, all interviews were conducted via Zoom and lasted about 45 minutes. Three student researchers previously trained in qualitative data collection, ethics, and compliance were enlisted to facilitate the interviews. Student researchers verified that the informed consent and intake survey were completed prior to the start of the interview, and using the intake survey, we were able to capture demographic data from our sample. The interview protocol consisted of open-ended questions adapted from a similar study surrounding the initiation of pre-exposure prophylaxis amongst Black men who have sex with men [50]. Two versions of the interview protocol were developed based on vaccination status. If participants indicated that they were vaccinated, their interview focused on the facilitators/motivating factors that encouraged them to do so. On the other hand, if indicated that they were not vaccinated, interview questions focused on the barriers to completing the vaccine series. Following the completion of the interviews, student researchers accessed the Zoom transcript files and cleaned them for data analysis.

### Data Analysis

Descriptive statistics were computed for all variables using StataSE (version 14; StataCorp LLC). The total number of responses and percentages were reported for categorical variables; mean and standard deviation were reported for continuous variables.

Using NVivo software (QSR International), 2 researchers trained in qualitative data analysis and NVivo software technology coded the 47 interviews. Using the constant comparative method, a codebook was developed for thematic coding and analysis by the research team [51]. Additionally, to reduce bias and to establish interrater reliability, the researchers developed a schedule for coding and a coding scheme. Each week, the researchers would code 10% of the transcripts independently and then merge coding findings into one dataset. The research team would then meet to run a coding comparison query in NVivo. Any data set sample that reported an agreement less than 90% was discussed to ensure that each individual researcher understood the codes and associated definitions/applications. The data set was coded again by the researcher individually, and the process was replicated until 90%‐100% agreement was achieved. A coding agreement of 90%‐100% means that minimal to no disagreements were reported using kappa coefficient in NVivo and consensus was established [52]. After coding was completed, the data were organized using NVivo software to explore emerging patterns and themes.

### Positionality of the Research Team

The diverse racial, cultural, and professional backgrounds of the research team included Black Caribbean, Black US American, White US American, and Asian American. This diversity allowed for study participants to authentically express their thoughts and feel comfortable sharing their lived experiences. Four of the authors hold primary backgrounds in public health, with experiences in health equity, community-centered research, and systems-level changes that shaped the framing of research questions, participant response, and theme prioritization during the qualitative analysis, which enriched the study depth. The secondary author is a foreign-trained medical physician whose clinical experiences in low-resource settings shaped a unique understanding of the structural inequities within health care and the importance of the patient-physician relationship, allowing for a deeper contextual understanding of participant responses.

Furthermore, each author’s unique personal experiences shaped how we approached the research process, and we acknowledge that our diverse professional and cultural contexts may have introduced potential biases within the analysis. To avoid speaking for the data, the team used reflexivity and iterative discussions. This included note-taking, which allowed the team to document and bring to light preconceptions while simultaneously ensuring that our interpretations were grounded in participants’ voices. The team sought to ensure that our research findings were both ethically grounded and accurately interpreted through collaborative discussion and critical reflection.

## Results

### Quantitative Findings

Although initially 60 participants signed up to participate, only 47 total interviews were conducted, resulting in a 78.33% completion rate. Demographic characteristics observed within our cohort were as follows: 51.1% (24/47) of the cohort were 27 years and older, and 48.9% (23/47) of the participants were White (Table 1). The breakdown concerning gender identity were as follows: 44.7% (21/47) of the participants identified as a man, 36.2% (28/47) of the participants identified as a woman, 6.4% (3/47) of the participants were nonbinary, and 4.3% (2/47) of the participants were gender fluid. A total of 29.8% (14/47) of the participants of our sample identified as gay, 19.1% (9/47) of the participants were bisexual, 14.9% (7/47) of the participants were lesbian, 12.8% (6/47) of the participants were pansexual, and 8.5% (4/47) of the participants were queer. In addition, 53.2% (25/47) of the participants indicated that they were single, and 19.1% (9/47) of the participants indicated that they were in a monogamous relationship. Around 74.5% (35/47) of the participants of our sample had been sexually active in the last 6 months, and 14.9% (7/47) of the participants engaged in unprotected sex of some kind. Thirty-six out of 47 (54.4%) participants indicated that their total lifetime partners were between 5 and 29 (54.4%) and 29.8% (14/47) of the participants never had a sexually transmitted disease. Forty-three out of 47 (91.5%) participants had not been diagnosed with HPV, and 28/33 (84.9%) participants tested indicated that they were HIV negative. Additionally, 22/47 participants (46.8%) indicated that they initiated the HPV vaccination.

**Table 1.** Demographic characteristics (N=47).

	Values, n (%)
Age (years)	
20	10 (21.3)
21‐23	8 (17.0)
24‐26	5 (10.6)
27 and older	24 (51.1)
Race and ethnicity	
Asian	2 (4.3)
Biracial or multiracial	6 (12.8)
Black or African American	14 (29.8)
Hispanic	2 (4.3)
White or Caucasian	23 (48.9)
Assigned sex at birth	
Female	28 (59.6)
Male	19 (40.4)
Gender identity	
Gender fluid	2 (4.3)
Genderqueer	1 (2.1)
Man	21 (44.7)
Nonbinary	3 (6.4)
Other or self-identify	3 (6.4)
Woman	17 (36.2)
Sexuality	
Asexual	2 (4.3)
Bisexual	9 (19.1)
Gay	14 (29.8)
Heterosexual	1 (2.1)
Lesbian	7 (14.9)
Pansexual	6 (12.8)
Queer	4 (8.5)
Questioning	1 (2.1)
Other or self-identify	3 (6.4)
Relationship status	
Cohabitating	4 (8.5)
Married or partnered	9 (19.1)
Monogamous relationship (only dating one person)	9 (19.1)
Single (not in a relationship)	25 (53.2)
Sexually active (Last 6 mo)	
No	12 (25.5)
Yes	35 (74.5)
Sexual behaviors (n=41)	
Protected anal sex	6 (12.8)
Unprotected anal sex	9 (19.1)
Unprotected oral sex	7 (14.9)
Unprotected vaginal sex	12 (25.5)
Protected vaginal sex	7 (14.9)
Lifetime sexual partners	
1 to 4	11 (23.4)
5 to 7	7 (14.9)
8 to 14	7 (14.9)
15 to 29	12 (25.5)
30 or more	10 (21.3)
Have you ever been diagnosed with an STD^a^?	
No	33 (70.2)
Yes	14 (29.8)
Have you ever been tested for HIV?	
No	14 (29.8)
Yes	33 (70.2)
Have you ever been diagnosed with HPV?	
No	43 (91.5)
Yes	4 (8.5)
Have you ever received any doses of the HPV Vaccine?	
No	25 (53.2)
Yes	22 (46.8)
How many doses of the HPV vaccine have you received? (n=22)	
1 dose	2 (9.1)
2 doses	5 (22.7)
All 3 doses	15 (68.2)
HIV Status (n=33)	
Confirmatory positive	5 (10.6)
Negative	28 (84.8)
What kind of insurance do you have?	
Insurance through employer	22 (46.8)
No insurance	4 (8.5)
Other	2 (4.3)
Private insurance	9 (19.1)
Public or government sponsored	8 (17.0)
Unsure	2 (4.3)

a STD: sexually transmitted disease.

Of our sample, 68.2% (15/47) of the participants indicated that they had completed the HPV vaccination series (3 doses received) and most 46.8% (22/47) of the participants had health insurance through an employer followed by 19.1% (9/47) of the participants having private insurance and 17.0% (8/47) of the participants being public/government sponsored (Table 1).

Risk for getting HPV (how susceptible they were to contracting the virus) was assessed using 5 statements on a 1 to 7 Likert scale (Table 2). When provided with the statement “I am likely to get HPV,” 27.66% (13/47) of our participants responded not sure, followed by 23.40% (11/47) of the participants who strongly disagreed. Overall, 29.79% (12/47) of our study sample strongly disagreed with the statement “I am at high risk for getting HPV” and 27.66% (13/47) of the participants indicated that they were not sure when presented with the statement “HPV would be a serious threat to the quality of my life.” A total of 29.79% (14/47) of the participants responded not sure to the statement “HPV would be a severe threat to my health and 27.66% (13/47) of the participants strongly agreed that “HPV would be a severe threat to my sex life.” Cumulatively, our sample responded with an average score of 18.73 out of a possible 35 for all 5 statements used to measure perceived risk (Table 3).

**Table 2.** Human papillomavirus (HPV) perceived risk among sexual and gender minorities (N=47).

	HPV would be a severe threat to my sex life, n (%)	HPV would be a severe threat to my health, n (%)	HPV would be a serious threat to the quality of my life, n (%)	I am at high risk for getting HPV, n (%)	I am likely to get HPV, n (%)
Strongly agree	13 (27.70)	7 (14.90)	11 (23.40)	5 (10.60)	4 (8.51)
Agree	12 (25.50)	10 (21.30)	8 (17.00)	3 (6.40)	4 (8.51)
Slightly agree	8 (17.00)	10 (21.30)	6 (12.80)	4 (8.50)	4 (8.51)
Not sure	9 (19.10)	14 (29.80)	13 (27.70)	9 (19.10)	13 (27.66)
Slightly disagree	1 (2.10)	3 (6.40)	4 (8.50)	4 (8.50)	2 (4.26)
Disagree	3 (6.40)	2 (4.30)	4 (8.50)	10 (21.30)	9 (19.15)
Strongly disagree	1 (2.10)	0 (0.00)	1 (2.10)	12 (25.50)	11 (23.40)

**Table 3.** Distribution of perceived risk of human papillomavirus (HPV) scores among participants. Higher scores indicate a greater perceived risk of HPV. Score ranges reflect grouped responses based on participants’ self-assessment. Percentages are based on the total sample (N=47).

Range	Values, n (%)
Less than 15	4 (8.5)
16‐19	15 (32)
20‐23	14 (29.7)
24‐27	7 (14.8)
28‐35	5 (10.6)

### Qualitative Findings

Participant interviews revealed that “Real Outcomes,” and “Accurate Representation” were the main components that should be considered for inclusion in an HPV documentary tailored for sexual and gender minority populations. Interviewees also revealed insights into their unique “Health Care Experiences” and the “Importance of using social media to disseminate information.”

#### Health Care Experiences

Participants discussed the quality of the health care they have received as well as their exposure to health information. Health care providers see a variety of patients daily and therefore have a higher possibility of exposing individuals to health information [53]. However, according to the accounts relayed by the participants, providers and health systems are doing little to take advantage of that opportunity. As one participant described, they received health information rarely, or only when they asked questions.

*As far as suburban Atlanta goes, it’s much more difficult, right? Because, whenever you go into doctors’ clinics or stuff sometimes there are pamphlets, but sometimes there’s just not that much information. It’s like a, if you ask them, you get information sort of basis. It’s not, you can- they’ll tell you, without asking.*

Participants’ expressions of the quality of care they have received from their health care providers varied (Textbox 1). Participants preferred to use LGBTQ + friendly health care providers and clinics, with some mentioning that the major barrier faced was race, not sexuality. This is exemplified in the quote below:

*Yeah, I try to go to, I think something basic, like STD testing or like a routine checkup, yeah I try to go to the queer friendly spaces or places that are queer-affiliated. Um, where, as if it goes like, if it seems like I can’t handle it in urgent care, um I still try to avoid going to X clinic, because every single time I’ve been there it’s been trash treatment. And just the big bill, so first I’ll start with if I can’t do it with urgent care or like queer-friendly spaces, I will just try a new hospital. Is my new thing so yeah. Only a stigma I’m working against you know just being black, even in a predominantly black city.*

Textbox 1. **Participant quotes on health care experiences (health information exposure and quality of care).**

Health information exposure:

- *Both at the clinic, and there’s a there’s a like a truck that comes by, I guess, where you can also do it certain days of the month, things like that. So, I feel like for on campus people, it’s a lot easier to get information, even if you’re not necessarily looking for it.*
- *It just kind of seemed a little ridiculous, like um because I don’t know much about healthcare, and I don’t know much about testing so to have to ask her was like, a lot because I didn’t even know what to ask for. I was just like; do you have any other tests? And she was like oh yeah, do you want to like get this test done, I was like yeah, are there any other ones? Like, I’ll get all of them done.*
- *I mean I access the, you know, I get messages through like the telehealth portals and things like that around, um, different things that I’m asking about, um. I don’t think I really got much. I mean, I remember picking up a couple of brochures here and there about like the HPV vaccine, and different stuff, and reading them at the clinic.*
- *I just felt like she, you know, she really talks about you know how I can prevent certain things like you know here [are] some resources for you to look at you know if you ever needed like that she you know she was the one that pointed me towards the health department if I ever need any like STI testing...overall she’s providing a lot of information about different things about you know how to stay healthy, in general...*

Quality of care:

- *I don’t like going to the doctor, I very much avoid it, that can be kind of hard to nail down. Just because I’ve just been rubbed so wrong by going in and just constantly having to either a) explain my whole history again, um b) it’s just obvious through their mood or tone that they don’t respect me, or see me, as you know. They’re just uncomfortable being around me. Like, I was you know it’s very hard. It’s like, I’m now at the point where it’s like, 'okay, the doctor has to prove to me that I can trust them’.*
- *It really depends on where I go and if I get lucky or not.*
- *I think the major differences that doctor is inside the city of Atlanta, so. Most doctors that I see that are inside the city of Atlanta, the likelihood kind of shoots way up that I’m going to have an okay experience, but the further outside of Atlanta I go, the more likely, I am just sort of you know, have to explain things that are unrelated to my being there.*

Many of the participants shared that they avoid visiting their doctor due to past negative experiences, as well as discussed how they felt their health care providers needed to earn their trust before they could be completely open with their health care needs. The participants’ experiences highlight the need to improve the consistency of high-quality health care.

#### Dissemination

The participants indicated the importance of using social media as a channel for disseminating DST products (Textbox 2). The information should be presented in a manner that does not overwhelm viewers and is easy to understand, so that viewers are left satisfied with what they have learned. Participants highlighted the need for information to be just enough that it was not overwhelming and provided key takeaways.

Textbox 2. **Participant quotes on dissemination (digital storytelling and use of social media).**

Digital storytelling:

- *I will say, those that dive into the more human and emotional side. You know, especially when you’re dealing with those [who] are being affected by certain things, and it kind of, kind of pulls you in.*
- *If you have it shorter, you can reach more people, but when you have it longer you have more of a chance to make a higher impact. So, I feel like there are definitely advantages to both, so if you actually did both, then you could use one as a segue into the other.*
- *I think I’d go stay away from doing like the you know, like the really sort of tragic/emotional music, I guess, if you’re trying to lessen the stigma um more of just probably something more like trying to normalize it, have people living like their daily lives and stuff maybe like just slice of life type thing.*

Social media:

- *Popular social media websites. Like, the most successful sort of like, videos and stuff I’ve ended up like, seeing I guess like for like vitalities purposes, like going viral and whatever is like. If I follow people on like, multiple social media platforms, where, even if I don’t, and I see that video pop up more than once, or that sort of article or whatever I’m way more likely to see it.*
- *I think it’d be better to if it was shorter too. Somehow get it on social media and just get make it somehow get it to be viral honestly. So, it’s got [to be] super catchy.*
- *Honestly, I would say number one is like, the Internet. Um, and like, social media in general, just because um, speaking of the context of growing up in the South, like I, the first place, that I learned to go to look for LGBT like information at all, let alone LGBT healthcare is the Internet.*

*Have it be like hey, this is what this is, this is what it can do and just present the facts in an easy to understand manner, where people don’t feel overwhelmed by the information and just laying it out for people in a way that they can understand that they don’t have to go and do all of their like you do the research for them, and you say like here, it is, and this nice little bundle so you can take it away...*

Participants describe how they are drawn towards human and emotional perspectives in what they watch, especially when it comes to stories involving life experiences. The participants’ overall suggestions for DST include the importance of presentation, length of the videos or film, and sharing true stories that express emotion and highlight humanity. In relation to modes of dissemination, one participant shared that much of the information they learned relating to the LGBTQ+ community was via web-based mediums. Social media connects people who are like them and provides a safe place for those who do not have a safe place in real life. Because of this reason, it proved to be an optimal channel identified by participants as seen below:

*I would say, social media is pretty big honestly. Like a lot of people who feel like they don’t have voices in real life, I think, are more willing or feel more comfortable reaching out through social media. And I think, from your point of view, as someone who wants to provide more education and information and stuff. I think social media would be a great platform.*

Creating something as simple as a few short videos that provide health information and keep the interest of the viewer, and make it easy to share across multiple platforms, can increase the number of views and potentially lead to the videos going viral.

#### Representation

Accurate representation of the LGBTQ+ community was another theme that arose during the interviews (Textbox 3). Participants noted that there is a substantial difference between the media’s prototypical LGBTQ+ persona and the actual modern-day lives that they live. Accurately capturing their reality would be vital to increasing their engagement. Several of them noted that when they saw themselves being represented in popular sitcoms and television shows, their interest in that show or character increased instantly. Furthermore, it was noted that this representation should also encompass what it looks like to live with HPV, as well as diversity in terms of cultural backgrounds and races, as described by one participant:

*I would say, you know include all different race of people. Um, and not just that one kind. Um, you know, mix it up a bit. You know, black, white, Afr- I mean, Chinese, Japanese, Korean, Asian you know, go outside the normal box. You know, all sizes um, male, female, transgender. Um, just like ready to go out there, all ages.*

Textbox 3. **Participant quotes on representation (race and accurate representation of being LGBTQ+ [lesbian, gay, bisexual, transgender, queer/questioning]).**

Representation:

- *We should be represented in a way that includes all races. All, I would say, like all guys and females we’re not all the same, and some people, you can’t even look at them and know that you know that they’re like gay or that they’re even sick, you know.*
- *I would say, you know include all different race of people. Um, and not just that one kind. Um, you know, mix it up a bit. You know, black, white, Afr- I mean, Chinese, Japanese, Korean, Asian you know, go outside the normal box. You know, all sizes um, male, female, transgender. Um, just like ready to go out there, all ages.*
- *I think it’s definitely important to include some race representation. Because most, most race, they’re probably going to be like ’Oh. this isn’t for me’ and just turn it off or, or not pay attention, but finding a way to connect, to, including ace representation. Um, and why it’s important to think about, even if you’re not sexually active.*

Doing so would provide a common experience that participants felt would allow them to foster a greater connection with the topic, in addition to providing increased optimism and hope for those living with the disease.

#### Messaging

Participants also noted the importance of highlighting real outcomes when speaking about HPV. Notable perspectives mentioned the need to demonstrate not only the prevalence of the disease but also to demystify the disease to the general population. Various complications can arise from being infected with HPV; from cervical cancer, anal and penile cancer to more benign problems such as warts.

*Someone who has lived through it, or is living through it, or had to care for someone who had it, etc, or someone who even had it advance all the way to, to ovarian cancer or you know however it may affect men. That would be definitely attention grabbing.*

According to the participants, including this wide range of problems would be engaging and allow the audience to graphically connect with the importance of being vaccinated. They also mentioned that having caregivers or family members share their viewpoints would also be beneficial to connecting with the intended audience (Textbox 4).

Textbox 4. **Participant quotes on messaging (importance of real outcomes).**

Real outcomes:

- *Look for somebody that can make a case for getting vaccinated, you know. As well, somebody that- that has it and has had a lot of problems with it. Oh, you know I’ve had to do, you know, I had HPV and it turned to cervical cancer and I had to you know have my cervix removed... You know, and now I can never have children, I can’t you know, whatever because of this, you know get vaccinated. And so, make it a little bit somebody that can talk kind of graphically, just like I just grabbed your attention with just that though.*
- *It’s not necessarily separate from you, um, like if you’re not sexually active, then I can see how that would be kind of separate from you, but it could still happen to someone that you care about or it could, if you’re sexually active it can definitely happen to you.*
- *So, it’s like if you can’t make that personal connection, if you can’t you don’t think anyone around you has it, you think like, oh well, it’s not a real problem, or like, yeah. So, I think that’s just confusing because, obviously, people do have it so there’s it’s more of like a silent thing, where people just aren’t talking about it. Yeah, so kind of connecting that and making people realize that people do have it, they just don’t talk about it would be nice, I guess.*

## Discussion

### Principal Findings

Our goal within this study was to identify what characteristics are essential to include in the development of HPV-related vaccination intervention for an LGBTQ+ audience and to identify dissemination methods that would ensure virality. This study has highlighted 2 key items to include in digital stories: real outcomes and representation.

### Comparison to Prior Work

Past research surrounding patient narratives has shown them to be a useful tool to gather information, communication, engagement, persuasion, and health behavior change [54]. DST expands upon the utility of patient narratives through its ability to focus on capturing the experiences of populations to share findings in an engaging manner through digital media [34]. However, the use of DST has some trade-offs with health care professionals arguing that the quality or accuracy of media-based DST is a major concern due to their lack of medical expertise [54]. Thus, the creation of any DST method for any behavioral intervention should allow for the opportunity to collaborate with health care professionals to ensure the provision of reliable, evidence-based medical guidance.

Results from our formative inquiry support the well-reported health disparities that the LGBTQ+ population frequently experiences, such as stigma and discrimination from health care providers [43-46]. Stigma and discrimination in health care settings undermine effective communication, which is essential for delivering quality care and fostering self-confidence. When comparing the communication and care quality between races, researchers found that Hispanic and Asian survivors reported poorer communication than White cancer providers, with Asians also reporting poorer care quality [55].

There is a pattern of discrimination, stigma, and lack of awareness among health care providers that has impeded LGBTQ+ populations from accessing health care services and has had a major impact on their health [41] resulting in less sexually transmitted disease screenings, inappropriate contraceptive counseling, less contraceptive use, and misinformation on sexual education and reproductive promotion. For example, studies have shown that lesbian women have lower use of sexual and reproductive health services, leading to significantly lower rates of pap smear testing [56]. Additionally, the Center for Disease Control has reported that 71% of new HIV infections seen were among gay and bisexual men in the United States in 2022 [57]. The use of DST within this population would allow for increased engagement and connections with others (ie, patients, advocacy groups, caregivers, health care professionals, and policy makers) that share similar lived experiences. Furthermore, through sharing their unique stories via this method, they can alter preconceived notions about their susceptibility/risk, attack misinformation concerning vaccinations, and reduce the health care disparities seen, thus improving the overall health of the LGBTQ+ population.

### Strengths and Limitations

Several limitations exist within the study. First, our sampling methodology could be a limitation [58]. We used voluntary convenience sampling to recruit study participants [59]. It is argued that this type of nonprobability sampling is not truly representative of the entire population, as the sample is likely to overrepresent or underrepresent certain groups and mainly targets persons with stronger opinions. Furthermore, this method has a low external validity and a lack of transferability to other populations [60]. Future research should consider partnering with community organizations during the recruitment period, thus diversifying participant groups and perspectives. Second, our cohort was concentrated within the Atlanta area. Atlanta has a unique demographic, cultural, and public health characteristic, which includes a greater exposure to public health campaigns that may influence overall health behaviors and beliefs. DST interventions conducted in other states/ countries may encounter alternative barriers or facilitators to HPV vaccination and thus may discover that other elements may need to be included.

A more in-depth analysis of the resulting themes could have yielded a deeper understanding of participant viewpoints and their implications. While we used a mixed methods research design, we failed to triangulate the data collection due to the practical constraints related to participant recruitment, such as patient and researcher availability and stringent COVID-19 protocols. As a result, the conclusions and insights from the participants were based on a single approach, thus limiting their validity. Additionally, as we did not collect or analyze data regarding differences in findings between vaccination status and other characteristics, which limited our ability to explore how the perceived risk of HPV may vary across subgroups. Future research should consider analyzing this area in order to not only provide a deeper understanding of the effect that individual factors may have on perceived risk but also provide insight into how to tailor public health interventions.

### Conclusions

The LGBTQ+ community is often misrepresented, underrepresented, or not at all represented in media, nor do they often feel safe and comfortable enough to share their experiences with others outside of their community. The use of DST methods involving real outcomes and accurate representation would bridge the gap created by stigmatization in order to improve HPV vaccination adherence and reduce health care disparities within this population.

## Abbreviations:

DST: digital storytelling
HPV: human papillomavirus
LGBTQ+: lesbian, gay, bisexual, transgender, queer/questioning
